# Considering challenges for the new Alzheimer's drugs: Clinical, population, and health system perspectives

**DOI:** 10.1002/alz.14108

**Published:** 2024-08-06

**Authors:** Sebastian Walsh, Richard Merrick, Richard Milne, Shirley Nurock, Edo Richard, Carol Brayne

**Affiliations:** ^1^ Cambridge Public Health University of Cambridge Cambridge UK; ^2^ Kavli Centre for Ethics, Science, and the Public University of Cambridge Cambridge UK; ^3^ Department of Neurology Donders Institute for Brain Cognition and Behaviour Radboud University Medical Centre Nijmegen The Netherlands; ^4^ Department of Public and Occupational Health Amsterdam UMC University of Amsterdam Amsterdam Netherlands

**Keywords:** Alzheimer's disease, dementia, epidemiology, population representativeness, public health

## Abstract

Recent approvals of amyloid immunotherapy drugs for early Alzheimer's disease (AD) have been highly controversial. In this piece, we consider challenges from the clinical, population health, and health systems perspectives to the role that the new AD drugs might be expected to play, now and in the future, in alleviating the morbidity caused by AD in the population. Clinically, short‐term effects are small, adverse events are frequent, treatment regimens are burdensome, and, crucially, long‐term effects are unknown. At a population level, there is always likely to be a trade‐off between breadth of access and magnitude of benefit for any given individual. At a health system level, roll out of treatment even for only narrowly‐defined patient groups will involve considerable resources to identify and treat eligible patients, with profound opportunity costs. Our considered view on current evidence is that there are challenges from each perspective to imagining a foreseeable future in which amyloid immunotherapy significantly alleviates AD morbidity at scale.

**Highlights:**

- Recent approvals of Alzheimer's drugs have met with excitement but also controversy.
- Trial effects are small, adverse effects concerning, and long‐term effects unknown.
- Results from trial cohorts may not generalize to broader, more complex patients.
- Significant resource requirements of eligibility assessment and drug administration.
- Use in “presymptomatic” populations is not supported by current evidence.

## 1 INTRODUCTION

Few fields are as topical and controversial as that of Alzheimer's disease (AD). AD is often quoted as causing 70% of the 55 million cases of dementia worldwide,^1^ but this represents a heterogenous group, and even the definition of what constitutes AD is hotly debated.^2^, ^3^, ^4^ Regardless, AD is associated with significant human suffering and societal costs, and effective therapeutic options are desperately desired by all.

Recent US licensing of amyloid immunotherapy agents has been highly newsworthy, and contentious.^5^, ^6^, ^7^ In this piece, we apply clinical, population health, and health systems lenses to consider challenges to the role that the new AD drugs might be expected to play, now and in the future, in alleviating the morbidity caused by AD in the population. We present the pros and cons of various options that arise from these therapies (Panel 1).

**FIGURE 1:**
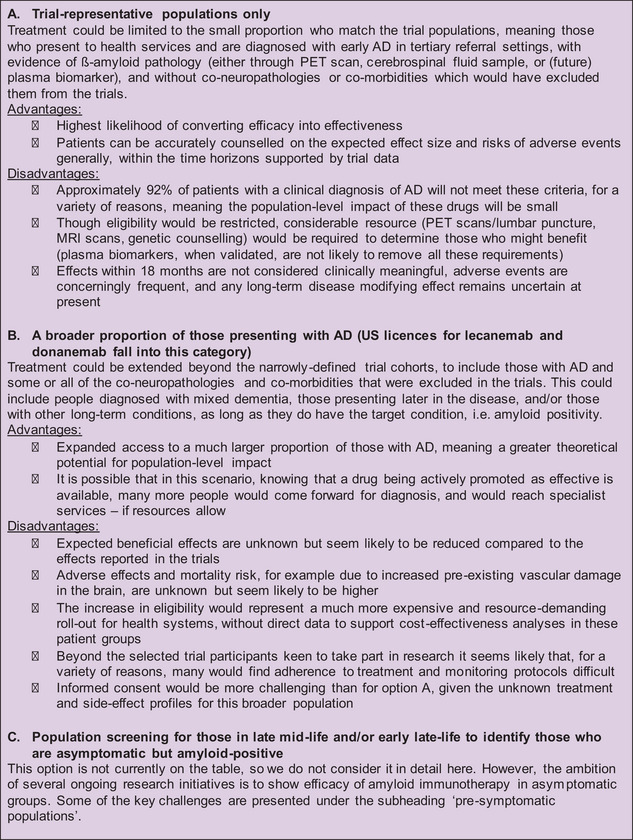
Panel 1—Options appraisal. With the aim of considering what role these therapies could play now (and in the future) to reduce population Alzheimer's morbidity, and with these three perspectives in mind, panel 1 presents scenarios of offering different groups of patients amyloid immunotherapy, with their pros and cons.

### 1.1 A clinical perspective

After two decades of trials reporting successful amyloid clearance but no resultant symptomatic benefit,^8^ recent years have seen two completed phase III randomized controlled trials of amyloid immunotherapy (lecanemab and donanemab) report statistically significant reductions in the rate of cognitive and functional decline compared to placebo.^9^, ^10^ However, the absolute effect sizes are small^11^ and clearly below previously established thresholds of the minimum clinically important difference.^12^ Moreover, only around 80% of participants completed the trials, and drop‐out was higher in the intervention groups than in the control groups.^9^, ^10^ This may have led to bias in favor of the intervention arms, even given the modified intention to treat analysis in the lecanemab trial which included > 95% of participants.

The drugs are also associated with significant adverse events, most seriously so‐called amyloid‐related imaging abnormalities (ARIA). Around 3 in every 10 people in the treatment arms experienced brain edema (ARIA‐E) and/or hemorrhage (ARIA‐H) (lecanemab 21.5%; donanemab 36.8%; placebo groups 9.5% and 14.9%, respectively). These abnormalities were imaging‐detected, with only one‐quarter of those with edema symptomatic, and few large hemorrhages, but the long‐term effects of these adverse events are unknown. Adverse events led to treatment discontinuation in 6.9% (lecanemab) and 13.1% (donanemab) of participants (placebo 2.9% and 4.3%, respectively).^9^, ^10^

Three deaths during the donanemab phase III trial (out of 853 people randomized to the treatment arm) were attributed to the treatment.^10^ There were no deaths attributed to lecanemab during the phase III trial.^9^ Two deaths during the open‐label extension of lecanemab have been described in detail.^13^, ^14^ One, a 65‐year‐old, died of extensive intraparenchymal hemorrhages following thrombolysis for ischemic stroke, occurring 4 days after the most recent lecanemab infusion.^14^ The other, the death of a 79‐year‐old, was attributed to severe amyloid‐related inflammation.^13^ In the donanemab trial, three treatment‐related deaths in the treatment arm occurred after serious ARIA.^10^

Infusion‐related reactions (IRRs) were also common in the trials (lecanemab 26.4%, placebo 7.4%; donanemab 8.7%, placebo 0.5%). This, along with ARIA, may have led to functional unblinding (participants and relatives correctly suspecting the trial arm). Functional unblinding is a particularly relevant concern for these trials, because many of the outcome measures, including the primary outcome measure, incorporated patient‐ and/or carer‐reports of symptom severity. The trialists undertook sensitivity analyses to try and account for this possibility, censoring participants after the occurrence of ARIA‐E (lecanemab)^9^ or ARIA‐E or IRRs (donanemab),^10^ and finding this had little effect on the primary effect size. However, by definition, these analyses censor those with side effects, most of whom are from the treatment arm and may therefore introduce an analytic bias in favor of the drugs.^5^, ^6^, ^15^ Results stratified by the occurrence of side effects were not reported. Functional unblinding therefore remains a concern that could challenge the internal validity of the trials, because it could explain some of the effect sizes reported.

The apolipoprotein E4 (APOE4) allele is an established genetic risk factor for AD. Up to two‐thirds of people with AD carry at least one APOE4 allele,^16^ and carriers typically have a faster progression of disease.^17^ It is therefore potentially concerning that subgroup analysis of the phase III trials suggests a reduced treatment response, and increased adverse event rate, in APOE4 carriers.^9^, ^10^ This suggests the need for genetic testing before starting treatment, with the associated additional burden to the health care system, including the need to provide appropriate counselling.^18^ Concerns have been raised that the trials were not powered to detect potentially important subgroup differences in effect sizes and adverse events by sex^19^ and ethnicity.^20^

The most crucial unknown is the long‐term effects of the drugs beyond the 18‐month trial period. The development of these drugs is oriented by the dominant *cascade hypothesis*, under which amyloid accumulation is thought to trigger a series of processes that together lead to dementia syndrome.^21^ Though the hypothesis is not universally accepted,^22^ the hope is that swift and near‐complete ß‐amyloid clearance will be disease‐modifying, with the effect accruing over time and ultimately leading to clinically meaningful slowing of decline.^23^ Indeed, those participants in the donanemab trial with low levels of tau pathology (i.e., earlier in the cascade) at baseline appeared to experience less decline than those with greater baseline tau who also received the drug, according to a prespecified subgroup analysis. Hypothesized long‐term, clinically meaningful slowing of decline can only be demonstrated by longer placebo‐controlled trials. The proposed phase IV observational registries are unsuitable to confirm benefits that differ from placebo, and more recently delayed‐start randomized trials have been proposed.^24^, ^25^

Despite all of the limitations, some people with early AD and their clinicians will choose to access these treatments where they are available. This is understandable given the lack of alternatives, and may be justified for some individuals, provided due care is given to shared decision‐making—this is made more difficult by hyperbolic reporting of relative effect sizes^11^, ^26^ and unfounded claims of “being able to drive for an extra 6 months”.^27^ Significant effort is needed to provide balanced information to patients and clinicians to support any such decision‐making process. This includes considerations about potential changes to patients’ existing treatment regimens to ensure eligibility, for example, stopping anticoagulants to account for the bleeding risk associated with starting the new AD drugs.

### 1.2 A population perspective

The trials included those with “early symptomatic Alzheimer's disease”—either mild dementia or mild cognitive impairment (MCI) with evidence of raised amyloid levels and, in the donanemab trial, additional evidence of raised tau. The MCI group, that is those with cognitive impairment but no accompanying functional limitation, was included because they represent the earliest symptomatic point in the hypothesized cascade and have a reasonably high likelihood of progression to dementia (a pooled analysis of 12 cohorts found that 59% progressed within 3 years,^28^ though selection biases in these cohorts mean this is likely an overestimate of the overall rate in the population).

The trials excluded those who had evidence of co‐neuropathologies (e.g., significant vascular pathology on MRI scan) which may have been contributing to their symptoms (minor co‐pathology was allowed), and those with co‐morbidities (e.g., depression, recent stroke, or cancer) which might increase the risk of trial noncompletion (Table 1). It is appropriate to set stringent eligibility criteria in a phase III trial designed to elicit *efficacy* (the best possible effect an intervention can achieve if conditions are set to its advantage). Nevertheless, it is important to consider who is represented in these trials in order to inform who should be offered these drugs if they are licensed (outside of the United States) and rolled out.

**TABLE 1:** Characteristics of participants in, and exclusion criteria for, the phase III trials of aducanumab, lecanemab, and donanemab

Parameter	Aducanumab	Lecanemab	Donanemab
Mean age (years)	70	71	73
Disease severity	80% MCI, 20% dementia Mean CDR‐SB 2.5	62% MCI, 38% dementia Mean CDR‐SB 3.2	17% MCI, 83% dementia Mean CDR‐SB 4.0
Comorbidities excluded (any)	Any condition (other than AD) that might be causing cognitive impairment. Stroke, TIA, unexplained LOC, in last year. MI, unstable angina, advanced CHF, conduction abnormalities, in the last year. Taking blood thinners. Alcohol or substance abuse, in the last year. Unstable psychiatric illness, in the last 6 months. Impaired renal or liver function. HIV positive. MRI/PET contraindications	Any condition (other than AD) that might be causing cognitive impairment. Stroke, TIA, or seizures, in the last year. Any nonadequately controlled bleeding disorder. Alcohol or substance abuse, within 2 years (known or suspected). Depression (GDS > 8). Any psychiatric diagnosis or symptoms that could interfere with study. Thyroid hormone abnormalities. Low B12. HIV positive, or any immunological disease requiring biological treatments. Malignant neoplasm, last 3 years. Severe visual or hearing impairment. BMI < 17, or > 35. Any other nonadequately controlled medical condition, or clinical abnormalities in physical examination, vital signs, lab tests, or ECG that the PI considers needs further management. MRI contraindications	Any condition (other than AD) that might be causing cognitive impairment. Alcohol or substance abuse, last 2 years. Any psychiatric disorder which may affect study analysis, any history of psychosis. Cancer in last 5 years. Hepatic impairment. Low literacy, visual impairment, or hearing impairment affecting neuropsychological testing. Multiple or severe drug allergies. Any serious or unstable medical condition, or clinical abnormalities in physical examination, vital signs, lab tests, or ECG, that the PI considers may affect study analyses. MRI/PET contraindications
Neurological co‐pathologies excluded	Acute or subacute hemorrhage, > 4 microhemorrhages, cortical infarcts, > 1 lacunar infarct, any superficial siderosis, white matter disease	>4 Micro‐ or any macro‐hemorrhages, > 1 lacunar infarct, any superficial siderosis, stroke involving a major vascular territory, severe small vessel or white matter disease, vasogenic edema, cerebral contusion, encephalomalacia, aneurysms, vascular malformations, infective lesions, SOL, brain tumors, or any MRI evidence that could indicate a non‐AD dementia	>4 Micro‐ or any macro‐hemorrhages, > 1 area of superficial siderosis, severe white matter disease, or any MRI evidence that could indicate a non‐AD dementia
% Eligible	25%	30%	21%

Abbreviations: AD, Alzheimer's disease; BMI, body mass index; CDR‐SB, Clinical Dementia Rating—the Sum of Boxes; CHF, chronic heart failure; ECG, electrocardiogram; HIV, human immunodeficiency virus; LOC, loss of consciousness; MCI, mild cognitive impairment; MI, myocardial infarction; MRI, magnetic resonance imaging; PET, positron emission tomography; PI, principal investigator; SOL, space occupying lesion; TIA, transient ischemic attack.

Press coverage of these drugs has implied relevance to anyone with a diagnosis of AD. But, for every 10 potentially eligible participants put forward for screening, 7 (lecanemab) or 8 (donanemab) did not meet trial eligibility criteria. Moreover, those considered for eligibility in these specialist research centers cannot be assumed to be representative of all patients with AD. Of those ineligible for the donanemab trial, one in four were excluded due to low amyloid levels (despite being considered potentially eligible for an Alzheimer's trial by recruiting clinicians), in keeping with data showing that a meaningful minority of those with a clinical diagnosis of AD do not in fact have raised amyloid.^29^

An analysis in the population‐based Mayo Clinic Study of Aging (MCSA) in the United States found that only 8% of those with MCI or mild dementia with raised amyloid levels met the eligibility criteria from the lecanemab trial.^30^ This is likely an overestimate of the generalizability of the trial cohorts to the total community‐based population with AD because of selection biases within the MCSA.^30^ Nevertheless, this is indicative of the highly selective nature of the trials and is unsurprising given the available population‐based evidence which shows a high prevalence of mixed dementia pathology and co‐morbidities. For example, an analysis of the National Alzheimer Coordinating Center (NACC) database in the United States found that, at the time of clinical AD diagnosis, 20% of patients were diagnosed with mixed dementia, 21% had clinical depression, and 5% had a history of stroke.^31^ Furthermore, the trials included participants with a mean age of 71 (lecanemab) and 73 (donanemab). In contrast, in the NACC database, the mean age at diagnosis of amnestic MCI was 80, and AD 85.^32^ Only part of this age gap can be explained by delays in presentation or diagnosis, further confirming the highly selective nature of trial populations compared to real‐world populations.^31^

The mismatch between trial cohorts, relatively younger with fewer co‐pathologies and co‐morbidities, and real‐world AD populations has significant implications for what effects we might expect to see if these drugs are rolled out more broadly. If a person's dementia is also determined by neuropathologies other than amyloid, which is particularly the case for older groups,^33^ it may be expected that they would have less to gain from the removal of amyloid compared to those in the trials who had a “purer” form of AD. People with cerebrovascular disease contributing to their cognitive impairment, also more common with age, may be more likely to experience established adverse events, most importantly brain hemorrhage. Older people and those with co‐morbid chronic diseases such as depression might find adhering to treatment regimens more difficult, including the requirement for carer attendance for treatment. Finally, the hypothesized long‐term benefits may be less likely to be realized for older groups given their relatively shorter life expectancy. Together, these considerations suggest that the more complex real‐world AD populations are less likely to achieve the same outcomes as trial participants, and are more likely to experience side effects.^34^

Concerns about translating trial efficacy into broader real‐world effectiveness are common to many diseases, increasingly so in the *“*precision medicine*”* era. Some current concerns may be alleviated over time, initial high drug prices may fall with competition, plasma biomarkers may reduce some imaging requirements, and subcutaneous preparations may replace intravenous infusions. However, two considerations of particular relevance to these AD drugs are likely to persist:

1. The effect sizes in the trials are very small. Despite efficient amyloid clearance early in the disease course and follow‐up for 18 months, trial completers still experienced most of the decline that the placebo group did (e.g., declining by 1.21 points instead of 1.66 points on an 18‐point scale^9^). Real‐world dilution of a trial efficacy, that is, already below estimated thresholds of minimum clinically meaningful effect^11^, ^12^ is unlikely to lead to population benefit.^17^
2. If meaningful long‐term benefits are demonstrated by extended placebo‐controlled trials, the assumption will remain that these drugs must be administered as early in the disease process as possible, in order to maximize their effect. Health services predominantly serve sick people. Drugs that must be given when symptoms are mild will always be more difficult to roll out effectively because people in these phases of disease are harder to identify.

### 1.3 A health system perspective

#### 1.3.1 Current approvals and likely clinical use in the near future

Controversially, in 2021 the Food and Drug Administration (FDA) first granted accelerated approval of aducanumab, another amyloid immunotherapy agent. Two parallel phase III trials were stopped early after meeting interim futility thresholds, but later re‐analysis of all data collected suggested statistically significant benefit in one of the two trials.^35^ Approval rested on amyloid removal as a surrogate endpoint, being *“*reasonably likely to predict clinical benefit.”^35^ Access was only granted via centers enrolled in a phase IV (or “postmarketing”) study—the value of which has been questioned.^36^, ^37^ Clinical use of aducanumab has been minimal, and one of the two‐phase IV trials has been abandoned due to low uptake.^38^, ^39^ In January 2024, the drug manufacturer Biogen announced that it was terminating its license to market Aducanumab in the United States.^40^

Lecanemab has been granted traditional approval by the FDA. The label states that lecanemab “is indicated for the treatment of AD. Treatment should be initiated in patients with MCI or mild dementia stage of disease, the population in which treatment was initiated in clinical trials.”^41^ However, the label does not contraindicate use in those who may meet these *inclusion* criteria but would have been *excluded* from the trials due to co‐neuropathology or co‐morbidity. Instead, there is a caution about offering lecanemab to patients with elevated intracerebral hemorrhage risk, though how exactly this might be measured and operationalized is unknown. Further, there is a “Black Box” warning about ARIA, in particular for APOE e4 homozygotes. Among other postmarketing requirements, the FDA has requested a registry‐based observational study to evaluate safety outcomes for those at increased hemorrhage risk (such as APOE e4 homozygotes and those with cerebral amyloid angiopathy).^42^ Further, Medicare has announced its own mandatory registry for all recipients of lecanemab.^43^

This means lecanemab has a label in the United States for a population that is beyond option A in our appraisal (Panel 1) and extends some way into option B. It is possible that the required cautionary labeling may lead to health systems, insurers, clinicians, and patients self‐regulating clinical use to those more closely reflecting trial populations, at least until more data are available. Donanemab was approved by the FDA in July 2024, with a similar label to lecanemab.^44^

In late July 2024, the European Medicines Agency (EMA) recommended the refusal of marketing authorisation for lecanemab, citing an unfavourable clinical risk/benefit ratio.^45^ The UK's Medicines and Healthcare Products Regulatory Agency (MHRA) is expected to make a decision on lecanemab imminently. If the MHRA do grant a license, then the UK's National Institute for Health and Care Excellence (NICE), which makes evidence‐based value‐for‐money judgements for the tax‐funded healthcare system, may find it difficult to approve such medications for use based solely on the limited trial outcomes. For example, NICE's evaluation scopes for these drugs^46^, ^47^ include mortality, ability to remain independent, and admission to full‐time care, for which evidence is currently lacking. Evaluation of longer‐term clinical outcomes continues to hinge on the predictive value of amyloid removal as a surrogate endpoint. But current evidence does not support this, a view shared by both NICE and the EMA.^48^, ^49^ Decisions from these regulators on donanemab are expected later in 2024.

#### 1.3.2 Resource requirements and value for money

The immunotherapy agents come with considerable resource requirements. Even if approved only for a small proportion of AD patients, the assessments required to identify the eligible group would include a much broader group of people. The logistics require careful thought. Would primary care physicians need to counsel and offer referrals to all patients with suspected MCI for specialist assessment? How will secondary services cope with this additional demand? Currently, eligibility assessment will require specialist clinical assessment, positron emission tomography (PET) scans or lumbar punctures to ascertain amyloid biomarker levels, MRI scans to confirm an absence of exclusion or caution criteria, and genetic testing to assess APOE4 status. Plasma biomarkers may replace some but not all of these requirements. Clinician time (already at a premium in over‐stretched health systems) would be required to counsel the eligible on the risk/benefit balance. Support would also be required for the large number of patients (up to 92% if eligibility is strictly limited to trial cohorts^30^) found to be ineligible, but who still receive an “early” diagnosis of AD, and who may perceive they have “failed” the eligibility tests for these much‐hyped new drugs. Those who are found to have insufficient amyloid to be eligible (which was one in four of those excluded in the donanemab trial) may request follow‐up assessments to determine eligibility in the future, and how this would be managed is unclear. The level of service redesign and consumption of health system resources required to successfully roll out these drugs will therefore produce “opportunity costs” (the benefits lost by not allocating these resources for other purposes) for all patients, including those with AD who do not choose to receive, or are ineligible for, the new drugs.

Those enrolled on drug treatment in the trials had to commit to attendance with a partner at an infusion center (the limited number of which initially would be likely to mean significant travel for some) every 2 weeks for lecanemab, or 4 weeks for donanemab. Subcutaneous preparations may reduce this burden if efficacy is demonstrated in ongoing trials. Serial MRI scans would be needed to monitor for adverse events, and health systems will require the resources to treat events as they occur.

If procedures from the donanemab trial are followed, and those who show sufficient amyloid clearance at periodic follow‐up assessments are taken off treatment, then routine monitoring may be required to inform recommencing treatment if/when amyloid re‐accumulates in the future. Evidence is needed to understand how often this monitoring may be required, and if it differs for different groups.

The debate on these drugs has focused almost entirely on high‐income countries. However, an increasing majority of dementia occurs in low‐ and middle‐income countries.^50^ Health systems in these countries are highly unlikely to have the resources required to consider eligibility for and offer these treatments, even to a very narrow group of those at high risk.

### 1.4 “Presymptomatic” populations—a future perspective?

A logical conclusion of the cascade hypothesis, in particular in the context of the modest reductions in decline reported in the trials despite highly effective amyloid clearance, is that “prevention” of AD can only be achieved by stopping the cascade before irreversible damage is done to the brain, and this means intervening before cognitive and functional deficits become apparent.^25^ Though not currently a policy option, this line of thinking is already embedded in current initiatives and trials.

A controversial 2018 US framework proposed a “biological” definition, labeling anyone with biomarker evidence of amyloid accumulation as being on the *“*Alzheimer's continuum*”* and those positive for amyloid and tau to have *“*Alzheimer's disease*”*, even in the absence of clinical symptoms of dementia.^3^ This was initially suggested only as a research framework, but triggered by the licensing of anti‐amyloid therapies in the United States, a 2024 update of this framework aims to “serve as a bridge between research and clinical care.”^51^ However, a recent 4‐year randomized trial of solanezumab in cognitively unimpaired people with elevated brain amyloid levels on a PET scan reported no clinical benefit.^52^ Another trial using lecanemab in an asymptomatic group with elevated amyloid, and either an APOE4 allele or a family history of dementia is ongoing.^53^

Asymptomatic ß‐amyloid pathology is prevalent, estimated as 3% in 50–59‐year‐olds, 18% in 60–69‐year‐olds, and 31% in 70–79‐year‐olds in one population‐based cohort.^54^ A key challenge would be how asymptomatic populations would be “screened” to determine eligibility. Aside from the logistics, modeling suggests that those with elevated amyloid levels but no symptoms or evidence of neurodegenerative change have a lifetime AD dementia risk of only 31% at age 60 (lower after that).^55^ How could we disentangle this group from the majority with no capacity to benefit? Considerations such as family history, genetic status, or biomarkers may assist risk stratification, and high‐quality epidemiological studies with minimal selection bias will be required to generate this evidence, including a thorough evaluation of the ethical implications. Such programs would have the clear capacity for potential net population harm, and could not be implemented on the basis of current evidence.

## 2 CONCLUDING THOUGHTS

Notwithstanding the field's collective desire for better therapeutic options for AD, our considered view on current evidence is that there are challenges from the clinical, population, and health system perspectives to imagining a foreseeable future in which amyloid immunotherapy significantly alleviates AD morbidity at scale. This view should be revisited if favorable data from appropriately conducted placebo‐controlled long‐term studies becomes available in the future. Uncontrolled phase IV studies will not provide these answers.

Clinically, short‐term reductions in cognitive decline are small, adverse events are frequent, and long‐term effects are unknown. Treatment regimens are burdensome on patients and their caregivers. At a population level, there is always likely to be a trade‐off in which any hypothetical future broadening of access will lead to a dilution of benefit for any given individual, due to the complexity of AD in real‐world populations. At a health system level, roll out of treatment even for only narrowly‐defined patient groups will involve considerable resources including personnel, with profound opportunity costs. This will be extremely challenging for even the best‐funded healthcare systems.

Previous decades have seen a field dominated by one theory, and a trial landscape dominated by one target. Based on current evidence, it is far from clear whether amyloid immunotherapy can ever significantly reduce population‐level dementia morbidity at scale.

## CONFLICT OF INTEREST STATEMENT

The authors declare no conflicts of interest. Author disclosures are available in the Supporting Information.

## Supporting information

Supporting Information
